# A Two-Stage YOLOv5s–U-Net Framework for Defect Localization and Segmentation in Overhead Transmission Lines

**DOI:** 10.3390/s25092903

**Published:** 2025-05-04

**Authors:** Aohua Li, Dacheng Li, Anjing Wang

**Affiliations:** 1 School of Mechanical Engineering, Hefei University of Technology, Hefei 230009, China; 2022180010@mail.hfut.edu.cn; 2 Anhui Institute of Optics and Fine Mechanics, Key Laboratory of General Optical Calibration and Characterization Technology, Chinese Academy of Sciences, Hefei 230031, China; dcli@aiofm.ac.cn

**Keywords:** transmission-line defects, defect localization, semantic segmentation, YOLOv5s, U-net, two-stage framework

## Abstract

Transmission-line defect detection is crucial for grid operation. Existing methods struggle to balance defect localization and fine segmentation. Therefore, this study proposes a novel cascaded two-stage framework that first utilizes YOLOv5s for the global localization of defective regions, and then uses U-Net for the fine segmentation of candidate regions. To improve the segmentation performance, U-Net adopts a transfer learning strategy based on the VGG16 pretrained model to alleviate the impact of limited dataset size on the training effect. Meanwhile, a hybrid loss function that combines Dice Loss and Focal Loss is designed to solve the small-target and class imbalance problems. This method integrates target detection and fine segmentation, enhancing detection precision and improving the extraction of detailed damage features. Experiments on the self-constructed dataset show that the method achieves 87% mAP on YOLOv5s, 88% U-Net damage recognition precision, a mean Dice coefficient of 93.66%, and 89% mIoU, demonstrating its effectiveness in accurately detecting transmission-line defects and efficiently segmenting the damage region, providing assistance for the intelligent operation and maintenance of transmission lines.

## 1. Introduction

The power system plays a fundamental role in modern economic and technological development. Among its components, transmission lines serve as a crucial channel for power delivery and must be regularly inspected to ensure safe and stable operation [1,2,3,4]. However, overhead transmission lines span long distances and are constantly exposed to natural environments, making them vulnerable to various climatic factors such as rain, snow, hail, and acid rain, as well as external forces. These conditions may result in surface damage (e.g., scratches and corrosion) and strand breakage, which can degrade the mechanical and electrical properties of the conductor and, in severe cases, lead to power outages or safety incidents [5,6,7].

In early stages, inspections mainly relied on manual visual checks by patrol personnel. With the development of UAVs, helicopters, and inspection robots, these technologies have been increasingly adopted in power-line inspection, significantly alleviating manual workload and improving efficiency [8,9,10,11,12]. However, defect recognition in transmission lines using these methods still largely depends on manual verification of the collected images, which is time consuming and prone to false detections or missed defects [13,14].

Before the widespread application of deep learning, defect detection in transmission lines mainly relied on traditional digital image-processing techniques [15]. These methods identified possible defects by analyzing changes in the shape of the wire, abnormal brightness, or texture discontinuities in the image. For example, [16] proposed an image-projection method based on grayscale variance normalization to detect surface damage and strand breakage; reference [17] suggested using Gabor filters to process video images for detecting broken strands; and [18] employed line structure extraction and local feature analysis to automatically identify strand breakage and foreign objects. These approaches effectively addressed some limitations of traditional transmission-line inspections, eliminating the need for manual image review and improving inspection efficiency and accuracy. However, they commonly suffer from poor adaptability to lighting changes and background interference, low robustness, and high rates of false or missed detections in complex scenarios.

With the rise in deep learning technologies [19], a variety of deep learning models based on object detection have shown promising performance in identifying and detecting defects [20,21,22,23], leading to their adoption in transmission-line inspection. These methods construct convolutional neural networks (CNNs) to automatically learn features from large volumes of images, enabling the accurate identification of defects such as strand breakage, foreign object intrusion, and surface damage without manual feature engineering. In [24], a YOLOv8-based detection model integrating visual and thermal imaging was proposed for power-line inspection. In [25], Faster R-CNN was employed to detect defects, foreign objects, and nested structures in wires. Reference [26] proposed an optimization strategy combining Context Information Enhancement (CIE) and Joint Heterogeneous Representation (JHR), which improved detection performance on small and occluded targets. In [27], YOLOv3 was used to achieve the real-time identification of intrusion targets. In [28], the YOLOv8n structure was optimized by incorporating the MSDA attention mechanism and Focal-EIOU loss function, significantly enhancing detection accuracy for line intrusions. In [29], a lightweight S_UNet was built based on U-Net [30], incorporating attention mechanisms and transfer learning for ice-covered conductor segmentation. A Generative Adversarial Network was further introduced to form SGAN_UNet, aiming to reduce prediction errors. However, most of these studies focus on component defects and intrusion detection, while relatively few address surface damage and strand breakage in the conductors themselves.

In research on transmission-line defect detection, reference [31] introduces data augmentation and lightweight network structures based on YOLOv7, combined with BiFPN feature fusion to improve detection accuracy and speed. Reference [32] proposes the ABG-YOLOv8n model, which integrates lightweight convolution structures, feature reconstruction mechanisms, and multi-scale enhancement to efficiently detect various defect types. Reference [33] introduces an adaptive threshold mechanism and GSConv structure to optimize the YOLOv8 model, improving transmission-line damage detection performance. Reference [34] presents a deep neural network called Fast Fusion Net, which enhances defect detection in complex scenarios through multi-feature fusion strategies. However, the above algorithms mainly focus on defect localization (bounding-box detection), lacking fine segmentation (pixel-level region extraction) of the damage area, which makes it difficult to meet the pixel-level information support required for subsequent maintenance or assessment.

To address this, some studies have shifted towards image segmentation-based methods for transmission-line defect recognition, performing pixel-level processing on the entire image to achieve more detailed region segmentation. For example, [35] implements wire breakage area segmentation based on DeepLab v3+, enhancing recognition performance through optimization algorithms. Reference [36] utilizes an improved U-Net model to achieve precise segmentation of various transmission-line defect areas. In comparison, while segmentation models can provide more precise defect boundary delineation, they face challenges such as insufficient sensitivity to small-scale defects and high computational costs for processing the entire image.

Therefore, this paper proposes a “detection-first, segmentation-second” two-stage strategy, which fully combines the fast localization capability of detection networks with the high precision characteristics of segmentation models, enabling the efficient and detailed identification of multi-class defects such as strand breakage and surface damage in transmission lines. The main contributions are as follows:

- A small-scale transmission-line defect dataset is constructed to address the lack of publicly available datasets. This self-built dataset includes a variety of typical defects in transmission lines, which are annotated to meet the requirements for model training and validation;
- A two-stage transmission-line defect detection framework based on YOLOv5s and U-Net is proposed. By combining the advantages of detection and segmentation, this framework achieves the collaborative processing of global and local image features. For strand breakage, characterized by high contrast and structural mutations, YOLOv5s is used for direct localization. For surface-damage areas with fuzzy edges and low contrast, U-Net is used for fine pixel-level segmentation after initial detection, enhancing the recognition accuracy of complex defects;
- Transfer learning and loss function optimization are introduced to improve model performance. YOLOv5s is initialized with COCO pretrained weights, and U-Net uses a VGG16 encoder pretrained on ImageNet as the feature-extraction module. A composite loss function combining Dice Loss and Focal Loss is constructed to alleviate issues related to class imbalance and small-target recognition;
- By comparing it with single detection or segmentation models, the proposed two-stage method demonstrates advantages in detection efficiency, segmentation details, and robustness, achieving improved defect-recognition precision and detail retention.

The remaining sections of this paper are arranged as follows: Section 2 provides a detailed introduction to the proposed detection framework and related optimization methods; Section 3 explains the data-processing flow, experimental design, and evaluates the model performance, while also discussing future improvement directions; Section 4 concludes the paper.

## 2. Methods

### 2.1. Model Structure of This Paper

**Figure 1:**
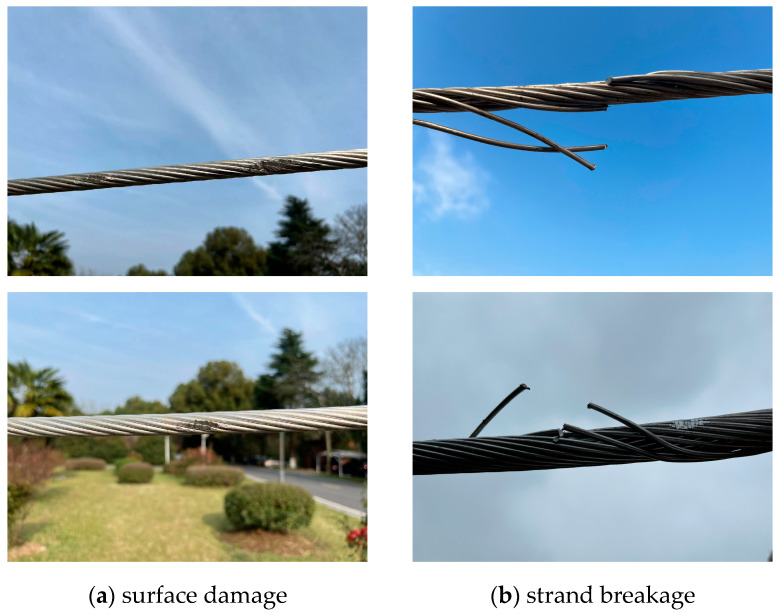
Schematic illustration of defect types. (**a**) Surface damage of transmission lines; (**b**) strand breakage in transmission lines.

In this study, transmission-line defects are categorized into two types: strand breakage and surface damage. Strand breakage refers to the physical fracture of the metal strands, typically characterized by high-contrast structural features such as abrupt geometric changes and texture discontinuities. In contrast, surface damage (e.g., corrosion, scratches) usually exhibits low-contrast characteristics with blurred or gradual edges, making it more difficult to detect. A schematic illustration of these defect types is shown in Figure 1. To address their differing characteristics, we adopt a task-oriented, stage-wise processing strategy in the model design. Given the distinct structural features of strand breakage, YOLO is used to directly detect and localize these regions. Moreover, the pixel-level segmentation of strand breakage adds limited practical value, so object detection alone is considered sufficient. Surface damage, however, tends to exhibit irregular boundaries and complex morphology. Therefore, after initial detection, a U-Net model is employed to perform pixel-level segmentation, enabling the precise extraction of damage edges.

To this end, we propose a hybrid architecture combining YOLOv5s and U-Net to detect and locate both surface damage and strand breakage in transmission lines. The main goal is to leverage the strengths of object detection and image segmentation, thereby enhancing overall performance in defect analysis. YOLOv5s rapidly filters potential defect regions, reducing the computational burden for U-Net, which focuses on the fine segmentation of local areas. This approach avoids global redundancy while preserving detailed boundary information of the damaged regions.

#### Overall Framework Design

**Figure 2:**
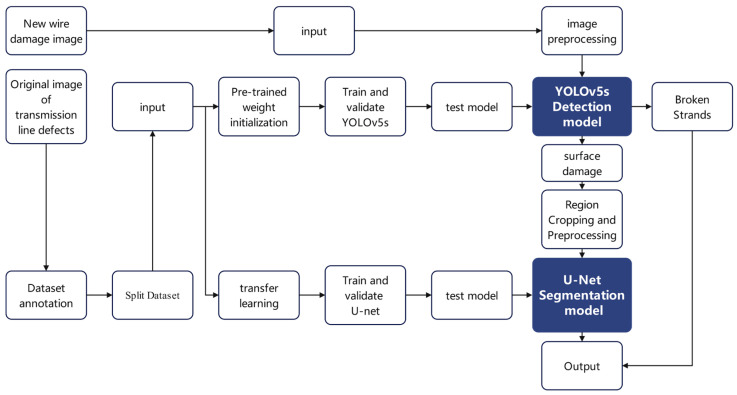
Two-stage detection framework structure based on YOLOv5s and U-Net.

A two-stage detection framework is proposed in this study, consisting of a YOLOv5s-based localization module and a U-Net-based segmentation module to perform a progressive analysis of conductor defects. As shown in Figure 2, the process includes the following key steps:

- Global defect localization: The original inspection image is fed into the YOLOv5s network, which outputs bounding boxes and confidence scores for potential defect areas, and directly locates strand-breakage regions;
- Candidate region cropping: A relaxed confidence threshold (>0.25) is applied to maximize the capture of surface damage, including those with weak features. Lowering the threshold improves recall, ensuring that small or low-contrast defects proceed to the segmentation stage;
- Local fine segmentation: Cropped ROIs (Regions of Interest) are resized to a standard input size and passed into the pretrained U-Net model, which outputs pixel-level masks representing the exact shapes and edges of the defects.

### 2.2. Two-Stage Model Framework

#### 2.2.1. Global Detection

As a lightweight variant in the YOLO series, YOLOv5s contains only approximately 7.2 million parameters, resulting in low computational overhead. It adopts a single-stage detection architecture, enabling simultaneous bounding-box regression and classification in a single forward pass, which offers significant speed advantages. Moreover, its feature-extraction structure demonstrates strong perceptual capability for small-scale targets, making it well suited for identifying fine-grained defects such as strand breakage and surface damage [37]. The YOLO family has been widely applied in industrial defect detection tasks, showing robust and real-time performance in detecting subtle flaws [38], further confirming its applicability in complex inspection scenarios. Given its balance between speed and accuracy, YOLOv5s is adopted in this study as the backbone network for global defect localization.

**Figure 3:**
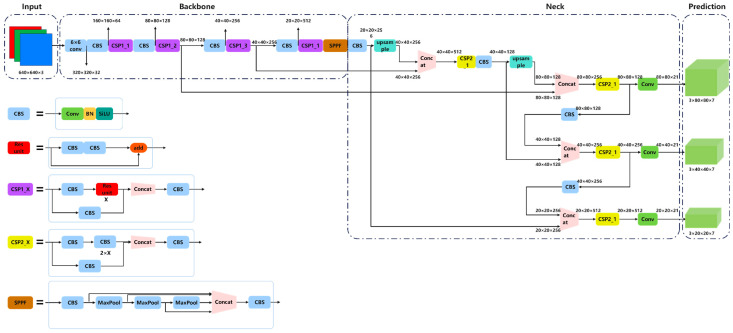
The architecture of the global detection model.

The YOLOv5s model developed in this study consists of an input layer, a backbone network (backbone), a feature fusion network (neck), and a prediction head (head). The relevant modules have been optimized to achieve efficient detection and localization of transmission-line defects. The architecture of the network is illustrated in Figure 3.

1. Input layer

The input layer is responsible for image preprocessing and augmentation, including resizing, normalization, and Mosaic data augmentation. Specifically, the input image is resized to 640 × 640 × 3, and the pixel values are scaled to the range of [0, 1] to meet the network’s requirement for value distribution. The Mosaic augmentation method randomly combines four images into one, generating more small targets at different locations and scales. This enhances the model’s ability to detect small-sized defects, such as conductor fractures and surface damage, by improving its sensitivity and robustness, particularly in detecting such small defects.

2. Backbone

The backbone network uses the CSPDarknet improved structure to extract multi-scale semantic features. In this study, a 6 × 6 convolution (stride 2, padding 2) replaces the original Focus module for initial downsampling and feature extraction. Compared with traditional slicing and concatenating operations, this method is more general and computationally efficient, effectively reducing computational overhead while enlarging the receptive field to preserve more low-frequency structural information.

The convolution is followed by a CBS (Conv + BatchNorm + SiLU) module, where Conv extracts local features, BatchNorm (BN) accelerates convergence and prevents gradient vanishing, and the SiLU (Sigmoid Linear Unit) activation function enhances non-linear expressiveness.

The core of the backbone network is the CSP module (Cross-Stage Partial), which divides the input feature channels into two parts. One part is directly connected, while the other part is passed into stacked residual modules and ultimately concatenated along the channel dimension. This structure effectively enhances gradient flow, reduces redundant computation, and increases feature reuse.

To improve the receptive field and multi-scale modeling ability, an improved SPPF (Spatial Pyramid Pooling—Fast) module replaces the original SPP. This module uses three sequential max-pooling operations (kernel 3 × 3, stride 1, padding 1) to build multi-scale features and concatenate them with the input feature map, enhancing the model’s perception of targets at various sizes and improving the discrimination of wire fractures and surface damages.

3. Neck

The Neck section uses the FPN (Feature Pyramid Network) + PAN (Path Aggregation Network) structure for multi-scale feature fusion and semantic information propagation. FPN propagates high-level semantic information down to the shallow layers to enhance small-target detection, while PAN supplements shallow structural information to high-level semantic features to compensate for spatial details. Multi-level feature fusion is achieved using nearest-neighbor upsampling and channel concatenation, outputting three feature maps at different resolutions (P3, P4, P5) for detecting small, medium, and large targets. This fusion mechanism strengthens the complementarity of shallow details (wire structure texture) and deep semantic information (defect categories), enabling the network to simultaneously identify macro structures and perceive micro surface defects, improving detection robustness in complex backgrounds and for different target sizes.

4. Head

The prediction head is based on the anchor mechanism, performing sliding window detection on feature maps at different scales. Each anchor outputs bounding-box offset, object confidence, and multi-class probabilities. The Sigmoid function is applied to normalize the classification and confidence branches.

To optimize the bounding-box regression process, the CIoU (Complete IoU) loss function is used for localization loss. CIoU further considers the center distance and aspect ratio differences between the predicted and ground truth boxes in addition to the IoU (Intersection over Union). The formula is as follows:

(1) $$L_{\mathrm{CIoU}}=1-\mathrm{IoU}+\frac{\rho^{2}\left(b,b^{\mathrm{gt}}\right)}{c^{2}}+\alpha v$$

Here, ρ is the Euclidean distance between the predicted and ground truth box centers, c is the diagonal length of the smallest enclosing box, and αv is the aspect ratio consistency measure.

The classification and confidence loss use binary cross-entropy (BCE) to enhance the network’s ability to discriminate between target and non-target areas.

(2) $$L=-\frac{1}{N}\sum_{i}^{N}\left[y_{i}\log\sigma \left(p_{i}\right)+\left(1-y_{i}\right)\log\left(1-\sigma \left(p_{i}\right)\right)\right]$$

where N represents the total number of samples involved in the calculation; y_i_ is the ground truth label of the i-th sample; p_i_ is the raw output of the model for the i-th sample; σ(p_i_) is the Sigmoid function, which compresses the value of pi into the [0, 1] range, representing the predicted probability that the object belongs to a specific class. It is defined as:

(3) $$\sigma \left(p_{i}\right)=\frac{1}{1+e^{-pi}}$$

The total loss function is:

(4) $$L=\lambda_{\mathrm{CIoU}}L_{\mathrm{CIoU}}+\lambda_{\mathrm{obj}}L_{\mathrm{obj}}+\lambda_{\mathrm{class}}L_{\mathrm{class}}$$

where λ is the weight coefficient.

Finally, the candidate bounding boxes predicted across different feature maps are aggregated and processed using Non-Maximum Suppression (NMS). NMS selects the bounding box with the highest confidence score and suppresses all other boxes that have an IoU above a predefined threshold with the selected box. This effectively eliminates redundant detections and ensures that each object is represented by only the most appropriate bounding box. Through this post-processing step, the model achieves accurate and reliable detection results, enabling a high-precision, end-to-end object detection pipeline.

To improve the model’s convergence speed and detection accuracy, this paper initializes YOLOv5s with pretrained weights based on the COCO dataset. The COCO dataset contains a variety of natural scenes and objects, and the model learns general low-level features such as edges, textures, colors, and spatial relationships during training on this dataset. The learned edge features help identify wire fractures, texture features enhance the perception of surface damages on wires, color and lighting features ensure stable detection in complex backgrounds, and spatial relationship features aid in understanding the connection between the wire structure and defects. By initializing the convolutional kernel parameters of YOLOv5s with pretrained weights, the network can quickly extract these low-level features when processing input images. Specifically, the initial convolutional kernel parameters have already learned basic visual features from the COCO dataset, helping the network recognize wire defects more effectively. As training progresses, the network fine-tunes these convolutional kernels through backpropagation to better adapt to the specific task, accelerating convergence and improving detection accuracy.

#### 2.2.2. Local Fine Segmentation

The surface-defect candidate regions generated by YOLOv5s are further refined using a U-Net model for high-precision image segmentation. U-Net, a convolutional neural network well suited for small-sample, high-accuracy segmentation tasks, employs a symmetric encoder–decoder architecture capable of effectively integrating multi-scale contextual information. It has been widely adopted in fine-grained segmentation scenarios such as medical image analysis. In recent years, U-Net has also demonstrated excellent performance in industrial defect detection tasks. For instance, an improved U-Net model proposed in [39] achieved high-quality segmentation of metal surface defects under small-sample conditions, further confirming its feasibility and effectiveness in this study. Therefore, U-Net is adopted as the backbone network for defect segmentation in this work. A transfer learning strategy is introduced by replacing the encoder structure, and a hybrid loss function is designed to improve both convergence speed and segmentation accuracy, resulting in promising experimental performance.

1. Transfer learning

In the U-Net model, this study introduces a transfer learning strategy to enhance training efficiency and generalization performance in small-sample scenarios [40]. Transfer learning is particularly effective when training data are limited but shares feature similarities with large-scale datasets. A model-based transfer learning approach is adopted, in which the VGG16 network, pretrained on the ImageNet image classification task, is used to initialize the encoder of the U-Net. The ImageNet dataset contains over 14 million images covering diverse natural scenes and structural patterns. The pretrained model’s mid- and low-level visual features (such as edges, textures, and shapes) can be effectively transferred to the target task in this study.

Specifically, the encoder part of U-Net is replaced by the VGG16 architecture, and its parameters pretrained on ImageNet are loaded as the initial weights. VGG16 consists of multiple 3 × 3 convolutional layers and max-pooling layers, which extract features hierarchically during downsampling. Max pooling reduces the spatial resolution of feature maps while increasing the number of channels, enabling richer feature representations. From conv1 to conv5, the feature map’s width and height are halved after each pooling operation, while the number of channels gradually increases. By the end of conv5, the feature map is reduced to 1/32 of the input size, with 512 channels, capturing high-level semantic features.

**Figure 4:**
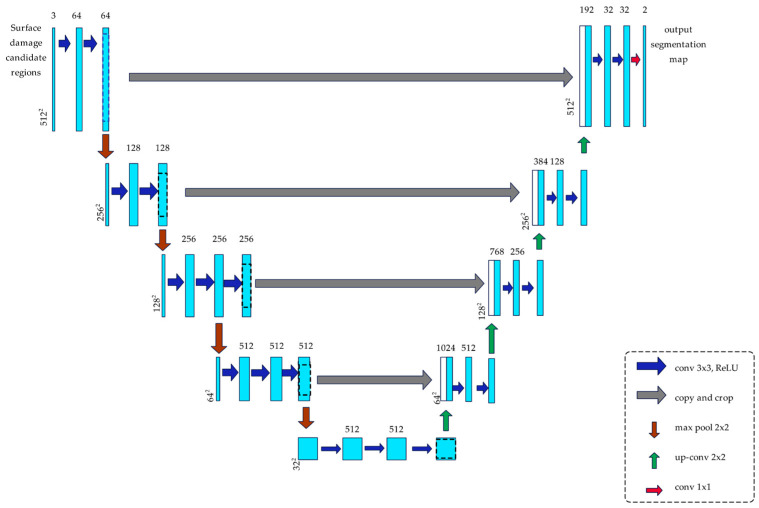
Framework diagram of the Vgg16-U-net model.

The decoder performs a progressive upsampling of feature maps using transposed convolutions and establishes skip connections with the corresponding encoder layers. This design enables the recovery of spatial resolution while integrating shallow-layer details with deep-layer semantics. After each upsampling step, 3 × 3 convolution layers are applied to refine features and reduce the number of channels. Finally, a 1 × 1 convolution maps the feature maps to the number of target classes, producing a pixel-wise classification probability map that matches the input size. The improved network architecture is illustrated in Figure 4.

2. Loss function

In this model segmentation task, the goal is to perform a binary segmentation of the surface damage on transmission lines. Since the proportion of background pixels is significantly higher than the target area, and there are issues with small targets and class imbalance, traditional loss functions like cross-entropy may not effectively address these challenges. Therefore, this paper combines Dice Loss and Focal Loss to better handle small targets and class imbalance.

Dice Loss aims to optimize the IoU of the predicted and real regions. This loss function is insensitive to object size, making it particularly suitable for small-object segmentation tasks. In cases where the background occupies a large portion of the image and the target region is small, Dice Loss effectively prevents the background from dominating the loss function, improving the segmentation accuracy of small targets. Its expression is shown in Formula (5). Focal Loss is an improvement based on the cross-entropy loss function, specifically designed to address class imbalance in pixel-level classification. Since the background class occupies the majority of the pixels in the image, traditional cross-entropy tends to ignore the target regions, negatively affecting segmentation results. Focal Loss focuses on hard-to-classify pixels, making the model pay more attention to small target areas, thus improving its ability to recognize small objects. Its expression is shown in Formula (6). Finally, by combining Dice Loss and Focal Loss with appropriate weighting, the total loss function is (7).

(5) $$\mathrm{dice}=1-\frac{2\sum_{i=1}^{N}p_{i}g_{i}+\varepsilon }{\sum_{i=1}^{N}p_{i}+\sum_{i=1}^{N}g_{i}+\varepsilon }$$

where P_i_ denotes the probability that the i-th pixel is predicted to be a positive class, g_i_ denotes the true label of the i-th pixel (0 is the background, 1 is the target), and ε is a smoothing term that prevents de-zeroing errors. N is the total number of pixels.

(6) $$\begin{array}{l} \mathrm{focal}=-\alpha {\left(1-p_{t}\right)}^{\gamma }\log\left(p_{t}\right)\\ p_{t}=\left\{\begin{array}{l} p_{i},\mathrm{if}\;g_{i}=1\\ 1-p_{i},\mathrm{otherwise} \end{array}\right. \end{array}$$

where α is the category balance weight, γ is the focusing parameter, and P_t_ is the model’s prediction confidence for the true category.

(7) total=λdice+(1−λ)focal

where λ is a weighting factor balancing the contribution of Focal Loss and Dice Loss.

## 3. Results and Discussion

### 3.1. Datasets

**Figure 5:**
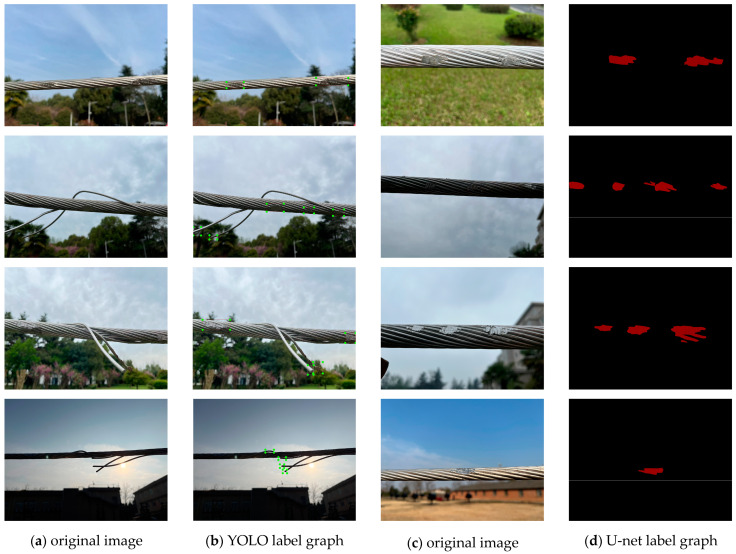
The sample transmission-line defect dataset. (**a**,**c**) The original images of transmission-line defects; (**b**) A label map of surface damage and broken strands corresponding to the YOLO model; (**d**) the corresponding binarized segmentation mask image of the U-net model.

Due to the limited availability of publicly available transmission-line defect images, a total of 673 original defect images were collected in this study. The imaging devices used include a digital camera and a Thorlabs scientific CMOS camera, covering various lighting conditions, weather scenarios, and background environments. Among these, the number of surface-damage defects is 1053, and the number of strand-breakage defects is 862. To enhance the model’s robustness and generalization ability, multiple data augmentation techniques were applied to the original images, including random rotation, horizontal flipping, brightness adjustment, blurring, and noise injection, ultimately expanding the dataset to 1241 images. All images are in JPG format, and each image may contain one or more types of defects, with an imbalanced distribution across categories. For object detection, we annotated bounding boxes using the Labelimg tool in VOC format, covering two defect categories: “surface damage” and “strand breakage,” which were used to train the YOLOv5s model. To enable high-precision segmentation, we further annotated surface-damage regions at the pixel level using the Labelme tool with polygon annotations, and converted the results into PNG-format binary masks, where the background is black and the defect regions are highlighted in red. To ensure annotation quality, all labeling was manually conducted by trained personnel and then verified through human cross-checking to eliminate errors and inconsistencies. Representative samples from the dataset are shown in Figure 5. Finally, the dataset was divided into training, validation, and test sets in a 7:1.5:1.5 ratio for model training and evaluation.

### 3.2. Experimental Environment

All model training in this study was conducted on the Kaggle platform using the PyTorch framework (Python 3.9), supported by an NVIDIA Tesla P100 GPU with 16 GB of VRAM. This hardware configuration met the memory requirements of both YOLOv5s and U-Net, and significantly improved training efficiency through GPU acceleration, ensuring stability and performance throughout the training process.

To enhance model performance, the YOLOv5s model was initialized with pretrained weights. These weights encode general low-level features such as edges and textures, which enhance the model’s ability to extract features relevant to transmission-line defect detection. In scenarios with limited training data, pretrained weights help the model converge faster and achieve better performance on the target task. During training, input images were resized to 640 × 640 to ensure accurate identification of surface defects and broken strands while reducing computational load and meeting GPU memory constraints. The batch size was set to 8, with a total of 100 training epochs. The initial learning rate was 0.001. The Adam optimizer was used due to its adaptive learning rate mechanism, which accelerates convergence on small datasets or under limited training epochs, while reducing the risk of oscillation or divergence.

In U-Net training, the encoder was built on a VGG16 model pretrained on the ImageNet dataset, enhancing feature representation and accelerating model convergence. Input images were resized to 512 × 512 to balance memory usage and edge-detail preservation. The optimizer was also set to Adam, and a composite loss function combining Dice Loss and Focal Loss was used to improve segmentation accuracy. A transfer learning strategy was adopted, involving two training phases: freezing and unfreezing. During the first 50 epochs (freezing phase), the VGG16 encoder was frozen to prevent the disruption of pretrained parameters at the early training stage. This strategy stabilized the training process and allowed the decoder to adapt more effectively to the new task, while mitigating the risk of overfitting caused by limited data. In this phase, the initial learning rate was set to 0.0001 to limit parameter update speed and protect the pretrained VGG16 weights. The batch size was set to 8, and only the decoder layers of U-Net were updated.

In the subsequent 50 epochs (unfreezing phase), the convolutional layers of the encoder network were unfrozen and included in training. At this stage, all model parameters were updated jointly to better meet the needs of high-level feature extraction for transmission-line defect detection. The learning rate was further reduced to 0.00001 to ensure that previously learned features remained stable during fine-tuning and to enable more refined parameter adjustments. The batch size remained at 8, ensuring consistent gradient distribution across both training phases, which facilitated the integration and overall improvement of model performance.

**Figure 6:**
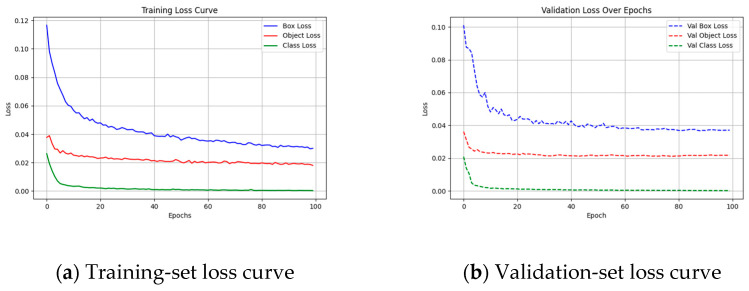
YOLOv5s model training loss curves. (**a**) Training-set loss curve; (**b**) validation-set loss curve.

The training results of the YOLO model are shown in Figure 6a illustrates the evolution of Box Loss, Object Loss, and Class Loss throughout training epochs. All three losses decrease rapidly in the early stages and then gradually stabilize, indicating good convergence. The Box Loss drops from an initial value of 0.12 to below 0.05, demonstrating the model’s capability in object localization. The Object Loss steadily decreases to around 0.03, suggesting improved confidence in target existence. The Class Loss approaches zero within the first 10 epochs, reflecting fast learning in category discrimination.

Figure 6b shows the validation loss curve, which is consistent with the training curve. The overall values are close, indicating that the model has not experienced significant overfitting, demonstrating strong generalization ability, and can stably handle complex transmission-line defect detection tasks.

**Figure 7:**
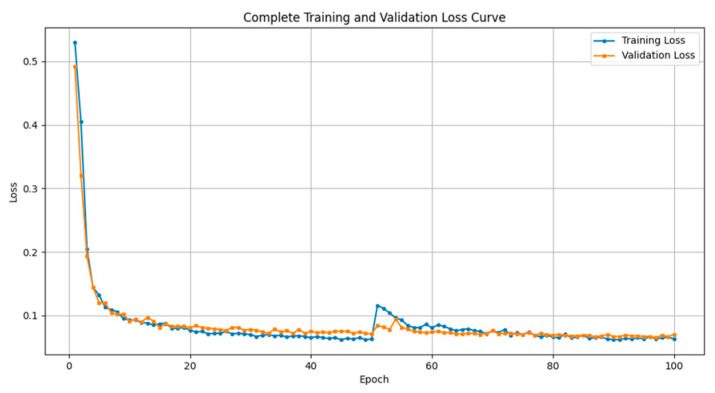
U-net model training loss curve.

Figure 7 shows the training and validation loss curves of the U-Net model. In the early training phase, the loss quickly drops from around 0.5 to below 0.1, indicating that the model can rapidly extract key features from the input images. The loss then continues to decrease more gradually and stabilizes after 60 epochs, reflecting good convergence and segmentation performance.

A brief fluctuation in the mid-to-late training phase may be attributed to the model’s adaptation to complex samples, such as images with blurred edges or strong background noise. Such localized disturbances are common during deep model optimization and did not disrupt the overall convergence trend. The model quickly returned to a stable state, demonstrating robustness. Throughout training, the training and validation loss curves remained close and consistent, indicating that no overfitting occurred and that the model possesses solid generalization capability.

### 3.3. Evaluation Indicators

Both YOLOv5s and U-Net have different evaluation metrics in target-detection and image segmentation tasks. In this experiment, YOLOv5s is used as a target-detection model, and we use recall, precision, and mean average precision (mAP) as model evaluation metrics.

1. Recall

Recall measures the proportion of all real targets that are successfully detected by the model, indicating the model’s sensitivity and its ability to avoid missed detections. The formula for this is

(8) $$\mathrm{Recall}=\frac{TP}{TP+FN}$$

where TP is the number of correctly detected targets and FN is the number of missed targets.

2. Precision

Precision measures the proportion of all boxes predicted by the model to be targets that are actually targets, reflecting the model’s ability to minimize false positives. The formula for this is

(9) $$\mathrm{Precision}=\frac{TP}{TP+FP}$$

where FP is the number of false-positive targets.

3. mAP

The mAP is the most frequently applied metric in the target-detection field measuring average precision for all categories, providing a comprehensive measure of overall detection performance. The formula for this is

(10) $$mAP=\frac{1}{N}\overset{N}{\underset{i=1}{\sum }}AP_{i}$$

where average precision (AP) is the average precision for each category, and AP is obtained by calculating the precision–recall curves at different IoU thresholds. mAP is the average of the AP values for all categories. N is the number of categories and $AP_{i}$ is the AP for each category.

When U-Net is used for image segmentation tasks, the performance is evaluated by Dice coefficient (DC), mean Intersection over Union (MIoU) and precision (P).

1. Dice Coefficient;

The Dice coefficient is a metric to evaluate the performance of a model by calculating the similarity between the predicted and real segmented regions, which is especially suitable for the case of unbalanced datasets. It is especially sensitive to small structures and balances false positives and false negatives. Its formula is as follows:

(11) $$DC=\frac{2|A\cap B|}{\left|A|+|B\right|}$$

Here, A represents predicted positive sample area (prediction mask), B is the true positive sample region (true mask).

2. MIoU

MIoU is an average of the Intersection over Union for each category, which reflects the overall performance of the model. It is calculated as

(12) $$MIoU=\frac{1}{k^{\prime }+1}\overset{k^{\prime }}{\underset{i=0}{\sum }}\frac{TP}{TP+FP+FN}$$

where $k^{\prime }$ denotes the number of categories, TP is the pixel correctly predicted to be in the positive category, FP is the pixel incorrectly predicted to be in the positive category, and FN is the pixel incorrectly predicted to be in the negative category.

3. Precision

Precision in image segmentation represents the fraction of pixels predicted as positive that are truly positive, highlighting the model’s ability to avoid over-segmentation and control false alarms. The formula is

(13) $$P=\frac{TP}{TP+FP}$$

### 3.4. Analysis of Experimental Results

In this section, various performance metrics of the model are analyzed to verify the applicability and performance of the proposed algorithmic framework in transmission conductor inspection scenarios. By evaluating the key metrics for the target-detection and semantic segmentation tasks, we explore the robustness of the model under different environmental conditions. Meanwhile, the effectiveness of the model in detecting defect locations and segmenting damage regions is analyzed through visualization results in order to comprehensively assess its potential for application in smart power inspection.

#### 3.4.1. Target-Detection Performance Analysis

**Figure 8:**
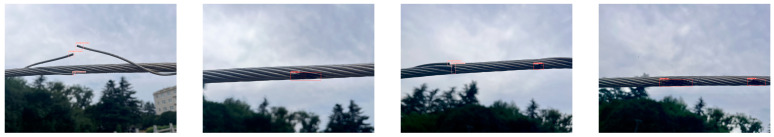

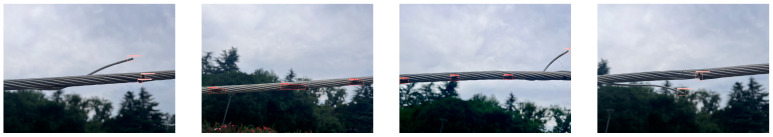
YOLOv5s model target-detection effect.

In the object-detection task based on the YOLOv5s model, the mAP reached 0.87, indicating the model’s strong overall recognition ability in multi-class defect detection. The precision was 0.89 and the recall was 0.85, demonstrating that the model effectively reduces false positives while accurately identifying most defect regions, showing good balance and robustness. As illustrated by the visual results in Figure 8, YOLOv5s is capable of accurately detecting and localizing both surface-damage and strand-breakage defects on transmission lines, further confirming its application potential in power-line defect detection tasks.

#### 3.4.2. Semantic Segmentation Performance Analysis

**Figure 9:**
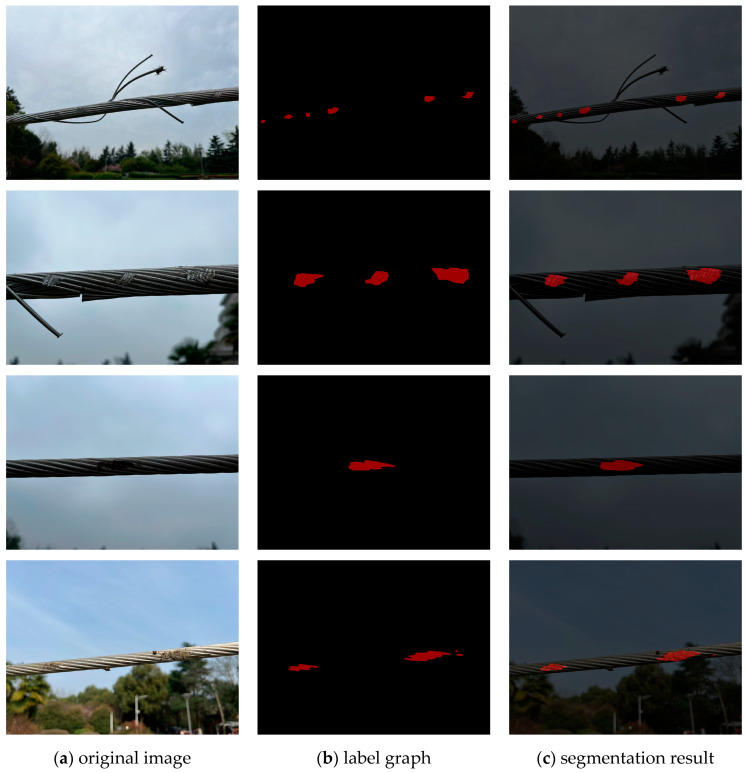
U-net model segmentation effect. (**a**) Original image of the dataset; (**b**) binarized mask label graph; (**c**) the results of the damage-area segmentation graph.

For the U-Net model, the segmentation performance is summarized as follows: the pixel accuracy of defect regions (P) reached 87.14%, the DC was 87.43%, with an average Dice of 93.66%, the overall pixel accuracy was 99.77%, and the mIoU reached 89%. These metrics collectively reflect the model’s accuracy and stability in delineating the contours of surface-defect regions, effectively avoiding issues such as under-segmentation or over-segmentation. As shown in Figure 9, U-Net successfully segmented complex surface defects on transmission lines, providing a reliable foundation for subsequent quantitative analysis of the damage.

#### 3.4.3. Model Advantages

This study focuses on the feasibility of a two-stage cascaded model based on YOLO and U-Net for transmission-line inspection scenarios. By leveraging the complementary strengths of both models, YOLOv5s is responsible for the fast detection of defect targets, thereby improving detection efficiency, while U-Net further performs fine-grained segmentation of surface-damage regions, enhancing the expression of damage edges and morphology. This combination enables the effective detection and precise segmentation of various types of transmission-line defects, providing pixel-level information for subsequent inspection tasks.

To further validate the model’s performance, we conduct a comparative analysis with the single-stage YOLOv5s and U-Net models on the test set. The performance comparison is carried out across three key metrics: precision, recall, and inference time, for the following reasons:

- Precision reflects the proportion of correctly identified defects among all detected instances. A higher precision indicates fewer false positives, which is critical for ensuring the safety of transmission systems;
- Recall measures the model’s ability to identify actual defects. A higher recall implies fewer missed detections, which is especially important for high-risk scenarios such as transmission-line monitoring to ensure that no defects are overlooked;
- Inference time directly affects the deployment efficiency and real-time applicability of the model. It is an essential metric to assess the system’s responsiveness and engineering viability in practical inspection tasks.

The comparison results are summarized in Table 1 below.

In terms of precision, the YOLOv5s single model demonstrates high detection accuracy, effectively reducing false positives. While U-Net has slightly lower precision, it offers pixel-level segmentation capabilities, allowing for the precise delineation of defect regions. The two-stage model proposed in this paper enhances precision by combining the fast object detection ability of YOLOv5s with the high-accuracy segmentation capability of U-Net, achieving higher-quality defect identification. In terms of recall, our model also outperforms both single models. It not only increases defect detection rates but also effectively controls false positives through the collaborative mechanism of the two-stage approach. Regarding inference time, the YOLOv5s single model, due to its lightweight structure, exhibits the fastest inference speed, making it suitable for rapid inspections. U-Net, on the other hand, has a longer inference time, making it more suitable for tasks requiring fine segmentation. In comparison, the two-stage model’s inference time is slightly higher than YOLOv5s but significantly reduces redundant computations compared to U-Net, balancing both efficiency and precision, making it highly practical for engineering deployment.

Overall, the two-stage model proposed in this study demonstrates stronger overall performance in transmission-line defect detection compared to the individual YOLOv5s and U-Net models. The combined model can efficiently perform defect detection and precise region segmentation, making it suitable for complex real-world application scenarios.

**Figure 10:**
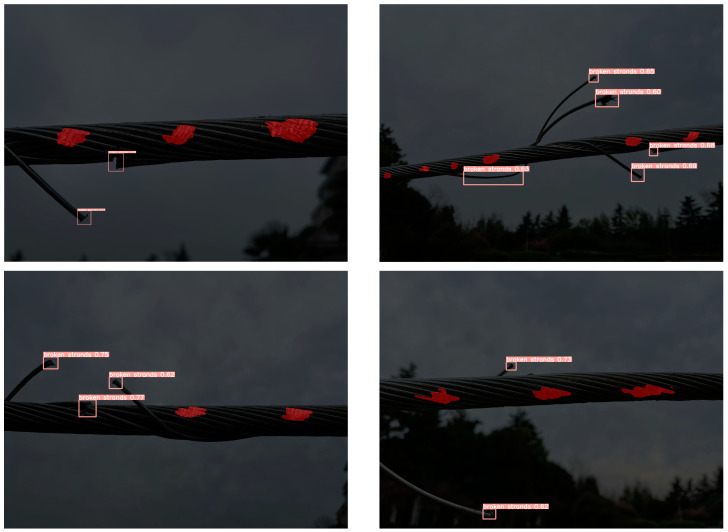
The model’s final detection result.

The original image of the transmission-line defects is input into the two-stage detection framework. First, the YOLOv5s model performs object detection and outputs the localization box for the broken chain area. Then, the ROI of the surface damage is input into the U-Net model for semantic segmentation. Figure 10 shows the final detection results, including the recognition box for the broken segment and the fine segmentation of the surface damage.

#### 3.4.4. Future Improvement Directions

Although the experimental results demonstrate good detection performance, there are still several areas that warrant further research and optimization. First, the diversity and scale of the dataset remain key factors affecting the model’s generalization ability. Future research could expand the power-line defect dataset to cover more defect types, thereby improving the model’s performance and generalization capacity. Additionally, future experiments should include comparisons with existing methods, aiming for better performance while maintaining feasibility. Finally, the model should undergo further lightweight optimization to meet the deployment requirements of embedded devices, enhancing its practicality and efficiency in smart power inspection tasks.

In addition, to more thoroughly assess the stability and reliability of the proposed approach, future work should pay closer attention to potential sources of error and bias that may affect detection performance. For example, environmental variations such as changes in weather, uneven lighting, and complex backgrounds during image acquisition may interfere with the model’s predictions. In rare cases, the model may still struggle with blurry boundaries or small-scale defects, which can be attributed to the limited number of such samples in the training dataset. Lastly, the proposed method has yet to be validated on larger-scale, cross-regional datasets, and its generalization ability across different scenarios warrants further investigation.

## 4. Conclusions

This paper proposes a two-stage cascaded framework based on YOLOv5s and U-Net for transmission-line defect inspection, addressing the challenges of insufficient accuracy and low efficiency in existing methods. In this framework, YOLOv5s first rapidly localizes defect regions, and U-Net then performs pixel-level segmentation on these candidate areas. By combining object detection with fine-grained segmentation, the approach effectively eliminates the redundancy of global computation and the lack of detail typical in traditional pipelines, thereby improving both accuracy and speed.

In the global localization stage, the YOLOv5s model is initialized with pretrained weights to enhance generalization. In the local segmentation stage, we employ an improved U-Net based on VGG16 and introduce a hybrid loss function combining Dice Loss and Focal Loss to achieve precise surface-damage segmentation.

The experimental results show that our two-stage framework achieves a precision of 0.91, a recall of 0.89, and an average inference time of just 0.08 s per image. These results demonstrate that the proposed method delivers high-quality defect identification efficiently, making it well suited for large-scale, real-time transmission-line inspections and providing support for intelligent transmission-line inspection.

## Figures and Tables

**Table 1:** Quantitative evaluation of single-stage and two-stage models.

Model	Precision	Recall	Inference Time (s)
YOLOv5s (Single)	0.89	0.85	0.05
U-Net (Single)	0.87	0.87	0.12
YOLOv5s + U-Net (Two-stage)	0.91	0.89	0.08

## Data Availability

The datasets used in this study can be accessed by contacting the corresponding author with a valid request.
